# *In vitro* Evaluation of Photodynamic Effects Against Biofilms of Dermatophytes Involved in Onychomycosis

**DOI:** 10.3389/fmicb.2019.01228

**Published:** 2019-06-07

**Authors:** Borui Chen, Yi Sun, Jinyan Zhang, Ruijun Chen, Xiurong Zhong, Xiaomo Wu, Libao Zheng, Jingjun Zhao

**Affiliations:** ^1^Department of Dermatology, Tongji Hospital, Tongji University School of Medicine, Shanghai, China; ^2^Dermatology Hospital of Fuzhou, Fuzhou, China; ^3^Department of Dermatology, Jingzhou Central Hospital, The Second Clinical Medical College, Yangtze University, Jingzhou, China; ^4^Electron Microscopy Laboratory, Fujian Medical University, Fuzhou, China

**Keywords:** dermatophytes, biofilm, onychomycosis, aPDT, dermatophytoma

## Abstract

Dermatophytes are the most common cause of onychomycosis, counting for 90% fungal nail infection. Although dermatophyte pathogens are normally susceptible to antifungal agents, onychomycosis often results in refractory chronic disease, and the formation of biofilms frequently underlines the inadequate responses and resistance to standard antifungal treatment. Numerous *in vitro* and *in vivo* antimicrobial photodynamic therapy (aPDT) studies have shown biofilm eradication or substantial reduction, however, such investigation has not yet been expanded to the biofilms of dermatophytes involved in onychomycosis. To shed a light on the potential application of aPDT in the clinic management of onychomycosis, in particular with the manifestation of dermatophytoma, we investigated photodynamic effects on the viabilities and the drug susceptibilities of the biofilm of dermatophytes *in vitro*. Here, methylene blue at the concentration of 8, 16, and 32 μg/ml applied as photosensitizing agent and LED (635 ± 10 nm, 60 J/cm^2^) as light source were employed against six strains of *Trichophyton rubrum*, ten strains of *Trichophyton mentagrophytes* and three strains of *Microsporum gypseum* isolated from clinical specimens. Our results indicated highly efficient photodynamic inhibition, exhibiting CFU (colony forming unit) reduction up to 4.6 log_10_, 4.3 log_10_, and 4.7 log_10_ against the biofilms formed by *T. rubrum, T. mentagrophytes*, and *M. gypseum*, respectively. Subjected biofilms displayed considerable decreases in SMICs (sessile minimum inhibitory concentrations) to multiple antifungal agents when compared with untreated groups, indicating the biofilms of dermatophytes became more susceptible to conventional antifungal drugs after aPDT. Additionally, the obliteration of biofilm after aPDT could be observed as shattered and ruptured structures being evident in SEM (Scanning Electron Microscopy) images. These findings suggest that aPDT is an attractive alternative treatment holding great promise for combating recalcitrant onychomycosis associated with the biofilm formation.

## Introduction

Onychomycosis is the most prevalent onychopathy that comprises 50% of nail disorders worldwide (Gupta et al., 2017). Dermatophytes are the predominant pathogens, followed by non-dermatophyte molds and yeasts responsible for approximately 10% of onychomycosis. Although dermatophytic pathogens are normally susceptible to antifungal agents, it is estimated that only 25–50% of patients with onychomycosis are cured after the standard treatment (Evans and Sigurgeirsson, 1999; Sigurgeirsson et al., 2002; Gupta et al., 2004; Baran et al., 2007). The presence of biofilm is considered to be a major contributing factor to the recalcitrance of chronic dermatophytic infection refractory to conventional antifungal regimes (Burkhart et al., 2002; Warshaw et al., 2005; Nusbaum et al., 2012; Costa-Orlandi et al., 2014).

Biofilm is a sessile microbial community in which microbes are embedded in highly compacted self-produced matrix of extracellular polymeric substances (EPS), composed of polysaccharides, proteins, extracellular DNA, membrane vesicles, etc (Al-Fattani and Douglas, 2006; Martins et al., 2010; Rajendran et al., 2013). The formation of biofilm is crucial for the microbial survival, sheltering microbes from a variety of environmental assaults, such as desiccation, UV-irradiation, antibiotics, and host immune system (Ceri et al., 2001; Ramage et al., 2012; Costa-Orlandi et al., 2014). Comparing to free-floating planktonic cells of the same species, biofilm pathogens can tolerate as much as 1000-fold higher levels of antimicrobial agents (Hawser and Douglas, 1995; Donlan and Costerton, 2002; Marsh, 2004) and microbial biofilms thereby account for more than 60% of all fungal and bacterial infections in humans (Cieplik et al., 2018).

Antimicrobial photodynamic therapy (aPDT) had been recently proposed to combat biofilms clinically (Lyon et al., 2011; Cieplik et al., 2014; Baltazar et al., 2015). As a non-antibiotic approach, aPDT employs non-toxic photosensitizers (PSs) and visible light at specific wavelength to generate reactive species of oxygen (ROS) and nitrogen (RNS), which are capable of killing microbes (Hamblin and Hasan, 2004; Alves et al., 2014; Taraszkiewicz et al., 2015). Notably, cytotoxic radicals produced by aPDT have extremely short half-lives and react only in their sites of formation, which reduces the cytotoxicity to adjacent normal tissues (Dai et al., 2009; Baltazar et al., 2015). Numerous *in vitro* as well as some *in vivo* aPDT studies have demonstrated aPDT has a broad-spectrum of activity against the biofilms and susceptible fungal pathogens and bacterial species include yeast (*Candida*. spp.), non-dermatophyte molds (*Fusarium* spp., *Exophiala* spp.), G^+^ bacteria (*S. aureus, E. faecalis*, and *Streptococcus* spp.) and G^-^ bacteria (*P. aeruginosa* and *Aggregatibacter. actinomycetemcomitans*) (Gilaberte et al., 2011; Junqueira et al., 2012; Seth et al., 2013; Beirao et al., 2014; Mannucci et al., 2014; Orlandi et al., 2014; Al-Ahmad et al., 2016; Gao et al., 2016; Carvalho et al., 2018).

However, the application of aPDT to biofilms formed by dermatophytes is less studied (Ali et al., 2016; Toukabri et al., 2018) and treatment with high efficacy remains challenging in clinic (Burkhart et al., 2002; Arrese and Pierard, 2003; Sigurgeirsson, 2010). Moreover, there are increasing rates of antimicrobial resistance among dermatophytes, especially for *Trichophyton rubrum*, the most frequent etiologic agent for onychomycosis (Baltazar et al., 2015). In an attempt to gain insight into the potential clinical implementation of aPDT tackling the dermatophytic biofilms implicated in onychomycosis, we investigated photodynamic effects on the viabilities, and the drug susceptibilities of the biofilm of dermatophytes, ranging from *T. rubrum, T. mentagrophytes* to *M. gypseum*.

## Materials and Methods

### Fungal Strains

Six strains of *T. rubrum* (Nos. 16463, 16355, 41452, 41467, 16618, and 41453), ten strains of *T. mentagrophytes* (Nos. 7240, 5614, 16446, 16339, 16494, 16077, MYA-4439, 8395, 8396, and 8397), and three strains of *M. gypseum* (Nos. 13789, 8305, and 8825) were supplied by the research center of medical mycology of Peking University (RCMMPU). All analyzed clinical isolates were collected from patients with onychomycosis and identified by molecular and morphologic methods. *C. parapsilosis* ATCC 22019 and *T.* mentagrophytes ATCC 4439 were included as control strains.

### Antifungal Agents

All antifungal drugs including terbinafine (TRB; purity ≥ 98%, SIGMA), itraconazole (ITC; purity ≥ 99%, SIGMA), cyclopirox (CLO; purity ≥ 99%, European Pharmacopoeia Reference Standard), and fluconazole (FLU; purity ≥ 98%, SIGMA) were purchased in powder form from Sigma Chemical Co., St. Louis, MO and prepared as outlined in the clinical and laboratory standards institute (CLSI) broth microdilution method M38-A2. The working concentration ranges of drugs were 0.0009785∼0.5 μg/ml for TRB, 0.03125∼16 μg/ml for ITC and CLO and 0.125∼64 μg/ml for FLU.

### Biofilm Preparation in 96-Well Microtiter Plates

Biofilm formation assay was performed in 96-well microtiter plates based on the method described by Costa-Orlandi et al. (2014) and further verified by Brilhante and Toukabri (Brilhante et al., 2017; Toukabri et al., 2018). The strains were grown on oatmeal agar (BD company) and incubated at 28°C for 14 days until sporulation. The inoculum was prepared by covering the cultures with 0.01M PBS (PH 7.2) adjusting to a final concentration of 1 × 10^6^ CFU/ml. Then, 100 μl of inoculum were added to 96-well plates (Corning 3599). The plates were incubated without agitation at 37°C for 3 h for biofilm pre-adhesion. Then, the supernatant was gently removed from the wells and the cells were washed three times with 0.01M PBS (PH 7.2) for removing non-adherent cells. Following that, 100 μl of RPMI 1640 medium were added and the plates were incubated at 37°C for 72 h. The media were then carefully extracted without disturbing the biofilm. The 96-well plate was washed with sterile PBS for three times to remove detached spores.

### Scanning Electron Microscopy

For SEM analysis, the preparation of biofilms was conducted on Thermanox coverslips (Thermo Fisher Scientific) instead of microtiter plates (Costa-Orlandi et al., 2014; de Aguiar Cordeiro et al., 2015). After 72 h incubation, the biofilms were fixed with 500 μl of 2.5% glutaraldehyde at 4°C overnight. Then biofilms were washed with cacodylate buffer twice, followed by 10 min dehydration with ethanol at each ascending concentrations (50, 70, 80, 95, and 100% ethanol) and drying for 30 min at 28°C. After drying, samples were dried in CO_2_, coated with gold and observed in a FEI Quanta 250 scanning electron microscope (FEI, Netherlands).

### Colony Forming Unit Counting

Colony forming unit is a cellular viability metric, measured by raw counts of clones growing in a standard sized Petri dish. Briefly, 200 μl of sterile water was added into each well of 96-well plate after the biofilm formation, followed by vigorous washing to thoroughly suspend the biofilm cells. The suspensions were then diluted 100 times by taking 2 μl of suspension diluted into 198 μl sterile water, after which half of the diluted suspension was used for inoculating a SDA plate for colony counting.

### XTT Reduction Colorimetric Assay

A semiquantitative measure of biofilm formation was calculated by using an XTT [2,3-bis(2-methoxy-4-nitro-5-sulfo-phenyl)-2H-tetra-zolium-5-carboxanilide] reduction assay, adapted from previous reports. XTT was prepared in a saturated solution at 0.5 g/liter in Ringer’s lactate. The solution was filter sterilized through a 0.22-μm-pore-size filter, aliquoted, and stored at -70°C. Prior to each assay, an aliquot of stock XTT was thawed, and menadione (Sigma; 10 mM prepared in acetone) was added to a final concentration of 1 μM. A 100 μl aliquot of the XTT-menadione solution was then added to each prewashed biofilm and to control wells (for the measurement of background XTT-reduction levels). The plates were then incubated in the dark for up to 2 h at 37°C. The activity of the fungal mitochondrial dehydrogenase reduces the tetrazolium salt XTT to formazan salts, resulting in a colorimetric change that correlates with cell viability. The colorimetric change was measured using an ELISA reader (Microplate Reader iMarkTM; BIO-RAD) at 490 nm. In all experiments, RPMI 1640 medium free of biofilm formation was included as a negative control (Mowat et al., 2007; Pitangui et al., 2012).

### Photodynamic Treatment

The PDI technique with modifications in the volume used, the incubation time, and the concentrations of methylene blue was described by Lyon et al. (2013). The methylene blue was tested at concentrations of 32 μg/ml (T1), 16 μg/ml (T2) and 8 μg/ml (T3), with 100 μl of each concentration added into 96-well plates containing biofilms. After incubation in dark for 3 h at 37°C, the biofilms were irradiated using a LED with an irradiance of 100 mW/cm^2^ at a wavelength of 635 ± 10 nm and a distance of 1 cm for 600 s (60 J/cm^2^). Control conditions were conducted as biofilms in PBS without irradiation (C1), biofilms with methylene blue (16 μg/ml) and without irradiation (C2), biofilms in PBS, and irradiated (C3).

### SMICs Determination

The values of SMICs were experimentally determined in this study using XTT-reduction colorimetric assay. The working concentrations of TRB, ITC, CLO, and FLU were prepared by a series of twofold dilutions (dilution range, 0.5–0.0009785 μg/ml for TRB; 16 to 0.03125 μg/ml for ITC and CLO; 64 to 0.125 μg/ml for FLU). The SMIC80s of TRB, ITC, CLO, and FLU were defined as the concentration at which 80% decrease in optical density would be detected in comparison to the mock controls in the absence of antimicrobial agents (Pierce et al., 2008). All tests were performed in triplicate.

### Statistical Analysis

Data were presented as the mean ± SD and analyzed with PRISM software package version 7.0 (XLSTAT Addinsoft, Paris, France). Three independent experiments were performed for all measurements. The differences between two groups were analyzed with Student’s *t*-test. Two-way ANOVA analysis was used to determine statistical differences among multiple groups. *p* < 0.05 was considered as statistical significance.

## Results

### Biofilm Morphology

**FIGURE 1:**
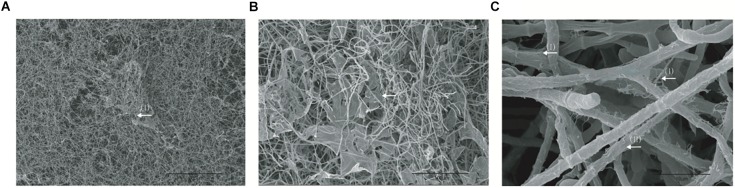
SEM images of the biofilms of *T. mentagrophytes* SEM provided three-dimensional images for biofilm structural assessment, with low magnification of x100 displayed in **(A)** and high magnifications of x500 and x5000 displayed in **(B)** and **(C)**, respectively. Two types of peculiar ECM architecture can be observed: (I) an extremely thin “blanket-like” layer covering the areas between hyphae **(B)**; (II) very fine “mesh-like” layer wrapping the filaments of hyphae **(C)**.

Biofilm formation using the strain of *T. mentagrophytes* 7240 islolated from clinical specimen was prepared on coverslip according to the method described by Costa-Orlandi et al. (2014). SEM providing three-dimensional images for in-depth structural assessment revealed that *T. mentagrophytes* 7240 produced noticeably robust biofilms with branched hyphae forming a mycelial network (Figure 1A), firmly attached to the coverslips. In particular, two types of peculiar extracellular matrix (ECM) architecture could be observed: (i) an extremely thin “blanket-like” layer covering the areas between hyphae (Figure 1B); (ii) very fine “mesh-like” layer wrapping the filaments of hyphae (Figure 1C). The biofilm morphology of high resolution and magnification was investigated and imaged by SEM technique to confirm the biofilm-formation in this study and the SEM images obtained are similar to those reported previously (Brilhante et al., 2017; Vila et al., 2017; Guzel Tunccan et al., 2018).

### aPDT Reducing the Viability of the Biofilms of Dermatophytes

**FIGURE 2:**
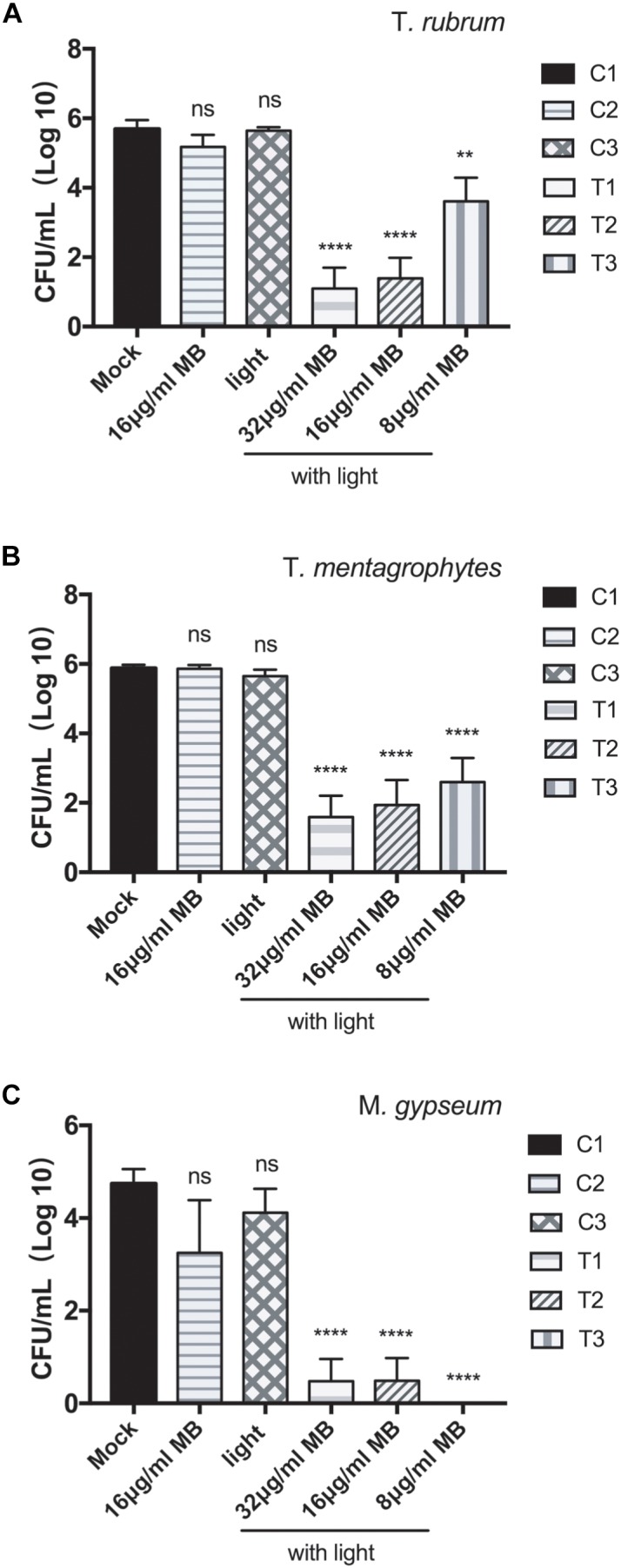
Photodynamic inhibition on the biofilms of *T. rubrum, T. mentagrophytes*, and *M. gypseum.* CFU counting indicated the viabilities of the biofilms of *T. rubrum* **(A)**, *T. mentagrophytes* **(B)**, and *M. gypseum* **(C)** under the conditions: T1, photodynamic treatment with 32 μg/ml of methylene blue; T2, photodynamic treatment with 16 μg/ml of methylene blue; T3, photodynamic treatment with 8 μg/ml of methylene blue; C1, mock condition with no irradiation and no methylene blue application; C2, exposed to 16 μg/ml of methylene blue without irradiation; C3, exposed to irradiation with no methylene blue applied. All data were represented as the mean ± SD (Two-way ANOVA, *p* values are in comparison to the results of C1 mock control: ^∗^*p* ≤ 0.05; ^∗∗^*p* ≤ 0.01; ^∗∗∗^*p* ≤ 0.001; ^∗∗∗∗^*p* ≤ 0.0001; ns *p >* 0.05).

The aPDT with LED (InGaAlP, 100 mW/cm^2^) exhibited CFU reduction by 2.0 log_10_, 4.3 log_10_, and 4.6 log_10_ against the biofilms formed by *T. rubrum* at the concentrations of MB 8, 16, and 32 μg/ml, respectively (Figure 2 and Table 1), demonstrating photodynamic inactivation in MB concentration dependent manner. The biofilms of *T. mentagrophytes* displayed the same pattern as to that of *T. rubrum*, with the CFU reductions at 3.3 log_10_, 4.0 log_10_, and 4.3 log_10_, accordingly. Interestingly, in contrast to *T. rubrum* and *T. mentagrophytes*, the CFU reduction of *M. gypseum* was more efficient at the concentration of MB 8 μg/ml (4.7 log_10_) than that of 32 μg/ml or 16 μg/ml (4.26 log_10_ and 4.25 log_10_, respectively). Such observation was probably due to generally high susceptibility of *M. gypseum* to aPDT and fewer strains have been tested in this study. Subsequently, no significant differences in CFU reductions were observed at the chosen concentrations of MB for *M. gypseum*. However, the efficiencies of aPDT against the biofilms of dermatophytes were lower than that of the biofilms formed by *Fusarium* spp., previously reported as 5.6 log_10_ in reduction with the same aPDT regimen (Gao et al., 2016), suggesting the biofilms of dermatophytes may be relatively more resistant to aPDT than other fungal pathogens associated biofilms. Nevertheless, with the CFU reductions ranging from 2 log_10_ to 4 log_10_, aPDT proved to be a highly effective approach against the biofilms of dermatophytes *in vitro*.

**Table 1:** Effects of photodynamic inhibition on the biofilms of dermatophytes.

Strain		Mock (CFU/ml)	16 μg/ml M (CFU/ml)	Light (CFU/ml)	32 μg/ml MB with light (CFU/ml)	16 μg/ml MB with light (CFU/ml)	8 μg/ml MB with light (CFU/ml)
*T. rubrum*	*RCMMPU-*16463	1.11 × 10^6^	7.67 × 10^5^	8.42 × 10^5^	5 × 10^3^	5 × 10^3^	2.02 × 10^5^
	*RCMMPU-*16355	9.75 × 10^5^	5.82 × 10^5^	7.7 × 10^5^	6.67 × 10^3^	5.5 × 10^4^	2.32 × 10^5^
	*RCMMPU*-41452	1.24 × 10^6^	1.64 × 10^6^	9.92 × 10^5^	0	1.67 × 10^3^	3.42 × 10^5^
	*RCMMPU*-41467	1.3 × 10^6^	1.33 × 10^6^	1.18 × 10^6^	2.83 × 10^5^	5.88 × 10^5^	8 × 10^5^
	*RCMMPU-*16618	4.5 × 10^4^	2.33 × 10^4^	2.45 × 10^5^	0	0	1.38 × 10^5^
	*RCMMPU-*41453	9.35 × 10^5^	3.25 × 10^5^	4.33 × 10^5^	0	0	6.67 × 10^3^
*T. mentagrophytes*	*RCMMPU-*7240	1.47 × 10^6^	1.87 × 10^6^	1.32 × 10^6^	1.43 × 10^5^	2.92 × 10^5^	4.43 × 10^5^
	*RCMMPU*-5614	1.96 × 10^6^	1.81 × 10^5^	1.48 × 10^6^	8.17 × 10^4^	1.15 × 10^5^	3.1 × 10^5^
	*RCMMPU-*16446	1.21 × 10^6^	1.13 × 10^6^	8.52 × 10^5^	4.17 × 10^4^	3.93 × 10^5^	5.53 × 10^5^
	*RCMMPU-*16339	1.1 × 10^6^	9.42 × 10^5^	7.78 × 10^5^	4 × 10^4^	1.5 × 10^5^	1.55 × 10^5^
	*RCMMPU-*16494	3.1 × 10^5^	6.07 × 10^5^	3.77 × 10^5^	0	0	0
	*RCMMPU-*16077	7.1 × 10^5^	5.53 × 10^5^	7.85 × 10^5^	0	0	0
	MYA-4439	1.6 × 10^6^	1.64 × 10^6^	1.09 × 10^6^	3.83 × 10^4^	3.87 × 10^5^	4.35 × 10^5^
	*RCMMPU-*8395	6.95 × 10^5^	6.33 × 10^5^	8.52 × 10^5^	0	0	1 × 10^4^
	*RCMMPU-*8396	3.08 × 10^5^	2.15 × 10^5^	6.07 × 10^5^	0	0	0
	*RCMMPU-*8397	3.62 × 10^5^	2.12 × 10^5^	3.3 × 10^5^	0	0	2.87 × 10^5^
*M. gypseum*	*RCMMPU-*13789	4.67 × 10^4^	1.67 × 10^3^	5.5 × 10^4^	0	0	0
	*RCMMPU*-8305	2 × 10^4^	7.67 × 10^4^	3.5 × 10^4^	6.67 × 10^3^	8.33 × 10^3^	0
	*RCMMPU-*8825	2.15 × 10^5^	1.88 × 10^5^	1.2 × 10^5^	0	0	0


*Data are mean values from three replicate experiments.*

### aPDT Increasing the Susceptibilities of Biofilms to Conventional Antimicrobial Agents

**FIGURE 3:**
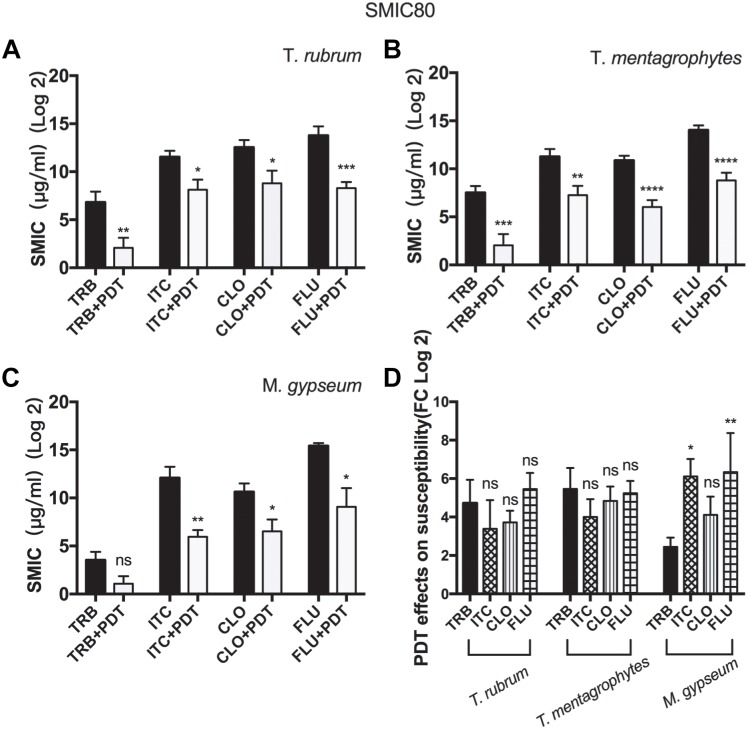
Photodynamic effects on the susceptibilities of dermatophytic biofilms to conventional antifungal reagents. The SMIC80 of the biofilms of *T. rubrum* **(A)**, *T. mentagrophytes* **(B)**, and *M. gypseum* **(C)** to TRB, ITC, CLO, and FLU were analyzed prior to or after photodynamic treatment. All data were represented as the mean ± SD [Unpaired *t*-test (one-tailed), ^∗^*p* ≤ 0.05; ^∗∗^*p* ≤ 0.01; ^∗∗∗^*p* ≤ 0.001; ^∗∗∗∗^*p* ≤ 0.0001; ns *p >* 0.05]. **(D)** Comparing how aPDT altered the susceptibilities of *T. rubrum, T. mentagrophytes*, and *M. gypseum* to TRB, ITC, CLO, and FLU (Two-way ANOVA, Tukey’s test, ^∗^*p* ≤ 0.05; ^∗∗^*p* ≤ 0.01; ^∗∗∗^*p* ≤ 0.001; ^∗∗∗∗^*p* ≤ 0.0001; ns *p >* 0.05).

SMIC ranges of terbinafine (TRB), itraconazole (ITC), cyclopirox (CLO), and fluconazole (FLU) against biofilms with or without aPDT (T1 regimen) were summarized in Figure 3 and Table 2. The susceptibilities of *T. rubrum, T. mentagrophytes*, and *M. gypseum* biofilms to these antifungal agents were variable, but TRB was consistently more efficient against fungal growth comparing to ITC, CLO, and FLE in all three species tested. The biofilms that were subjected to aPDT exhibited significant reductions in SMIC80 when compared with aPDT untreated groups, indicating that the treatment of aPDT effectively increased the susceptibilities of *T. rubrum, T. mentagrophytes* and *M. gypseum* to these conventional antimicrobial drugs. Furthermore, aPDT exerted comparable effects on increasing the susceptibility of *T. rubrum, T. mentagrophytes* to TRB, ITC, CLO, and FLU as shown in the Figure 3D. In contrast, the susceptibility of *M. gypseum* to TRB after aPDT was less affected, where the SMIC80 of TRB without aPDT was just four times higher than that with aPDT, much lower than 64-fold increase observed with ITC and FLU.

**Table 2:** Photodynamic effects on the SMIC80 of dermatophytic biofilms.

Strain		SMIC80 (μg/ml)							
	
		TRB		ITC		CLO		FLU	
				
		-PDT	+PDT	-PDT	+PDT	-PDT	+PDT	-PDT	+PDT
*T. rubrum*	*RCMMPU-*16463	0.146	0.022	3.000	0.042	16.000	6.667	64.000	0.708
	*RCMMPU-*16355	0.500	0.011	6.333	0.729	1.000	0.031	1.417	0.125
	*RCMMPU*-41452	0.012	0.006	1.833	4.000	13.333	1.333	48.000	3.333
	*RCMMPU*-41467	0.500	0.336	13.333	0.042	1.333	0.031	53.333	0.125
	*RCMMPU-*16618	0.500	0.001	9.333	2.010	12.000	1.677	22.750	0.250
	*RCMMPU-*41453	0.029	0.001	5.510	5.427	16.000	2.000	42.833	0.375
*T. mentagrophytes*	*RCMMPU-*7240	0.375	0.001	16.000	16.000	1.000	0.031	64.000	13.500
	*RCMMPU*-5614	0.500	0.001	16.000	5.750	1.000	0.031	25.333	0.125
	*RCMMPU-*16446	0.417	0.001	6.167	0.031	0.667	0.031	10.833	0.125
	*RCMMPU-*16339	0.500	0.002	6.833	0.385	16.000	0.031	3.667	0.125
	*RCMMPU-*16494	0.500	0.500	1.396	5.354	1.042	0.031	32.000	21.833
	*RCMMPU-*16077	0.500	0.500	6.167	5.354	8.667	0.031	48.000	42.708
	MYA-4439	0.500	0.011	11.000	0.031	1.750	0.031	43.333	0.458
	*RCMMPU-*8395	0.073	0.001	16.000	0.031	5.333	2.667	16.000	0.125
	*RCMMPU-*8396	0.027	0.001	0.229	0.031	2.167	0.042	64.000	0.125
	*RCMMPU-*8397	0.014	0.001	0.333	0.031	1.667	1.344	13.333	0.250
*M. gypseum*	*RCMMPU-*13789	0.172	0.084	16.000	0.698	6.708	0.031	42.667	24.333
	*RCMMPU*-8305	0.013	0.001	10.833	0.031	0.875	0.052	32.000	0.167
	*RCMMPU-*8825	0.005	0.001	5.542	0.063	8.167	1.021	64.000	0.125


*The methylene blue was tested at concentrations of 32 μg/ml as the regimen of T1. The biofilms were irradiated using a LED with an irradiance of 100 mW/cm^*2*^ at a wavelength of 635 ± 10 nm and a distance of 1 cm for 600 s (60 J/cm^*2*^). Data are mean values from three replicate experiments.*

### Obliteration of Biofilm Resulted From Photodynamic Therapy

**FIGURE 4:**
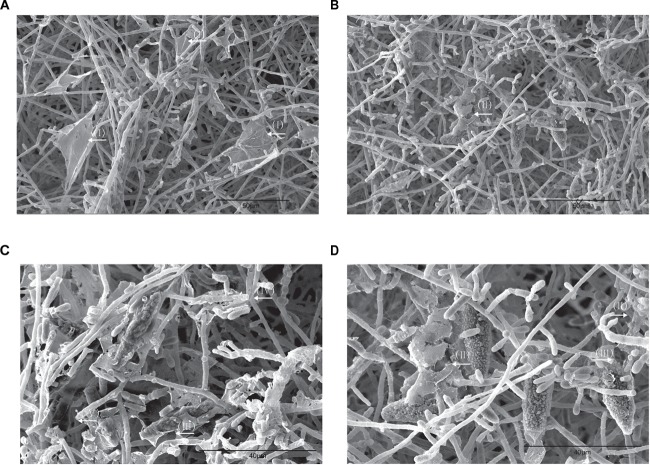
SEM images of the bioflms of *M. gypseum* after photodynamic treatment. **(A)** *M. gypseum* not subjected to photodynamic treatment. **(B–D)** *M. gypseum* subjected to photodynamic treatment. (I) Formation of a relatively complete membrane-like structure. (II) Biofilms appeared to have a “hole” in their surface, with a tearing appearance. (III) Perforated ECM surrounded the macroconidia. (IV) Mycelia were fractured, sections of hyphae were broken.

To investigate the morphological alteration of dermatophyte biofilm after aPDT (T1 regimen), SEM images of *M. gypseum* biofilm were obtained following the photodynamic treatment. As shown in the Figure 4, comparing to aPDT untreated specimen (Figure 4A) in which dense entangled hyphae exhibited regular morphology with uniform diameter and smooth surfaces as well as the characteristic “blanket-like” membrane and fine “mesh-like” wrapping layer were present, the mycelia of *M. gypseum* biofilm after aPDT treatment were fractured and shattered with raptured hyphae and fragmented “blanket-like” membranes (Figure 4B–D).

## Discussion

Onychomycosis is the most common nail infective disorder (Piraccini and Alessandrini, 2015) and is caused primarily by anthropophilic dermatophytes, in particular by *Trichophyton rubrum*, followed by *Trichophyton mentagrophytes* var. *interdigitale* (Faergemann and Baran, 2003). The non-dermatophyte molds, such as *Fusarium* spp., *Acremonium* spp., *Alternaria* spp., *Scopulariopsis brevicaulis, Aspergillus* spp., can also be involved in the pathogenesis with estimated 10% prevalence worldwide (Gupta and Nakrieko, 2014; Gasser et al., 2016; Gupta et al., 2016; Motamedi et al., 2016). Yeasts, like *Candida albicans* and *Candida parapsilosis*, represent the third cause of nail fungal infection, but only occurring when predisposing factors are present, mainly immunosuppression, and diabetes (Arrua et al., 2015; Gasser et al., 2016).

Developing novel therapeutic approach against the biofilms of dermatophytes implicated in recalcitrant onychomycosis presents a pressing need in the clinical management, especially the onychopathic condition with dermatophytoma (Sigurgeirsson, 2010). Initially established as a successful modality for malignancies and age-related macular degeneration (Dougherty et al., 1978; Orenstein et al., 1996), photodynamic inactivation has been shown as an effective alternative strategy for combating biofilms. The antimicrobial effects of aPDT have been observed on bacterial, non-dermatophytic, and yeast biofilms *in vitro* as well as *in vivo* using various animal models (Friedberg et al., 2001; Giroldo et al., 2009; Lyon et al., 2011; Soares et al., 2011; Takahashi et al., 2014; da Silva et al., 2018). However, the aPDT effect on dermatophytic biofilms has been less investigated due to the lack of reliable models. Fortunately, Costa-Orlandi et al. have recently successfully established the procedure to *in vitro* form the biofilms of dermatophytes using the stains of *T. rubrum* ATCC 28189 and *T. mentagrophytes* ATCC 11481 within 72 h, providing a valuable *in vitro* model to facilitate the investigation of photodynamic effect on dermatophytic biofilms (Costa-Orlandi et al., 2014). In addition to ATCC strains, the dermatophytic isolates obtained from clinical onychomycosis specimens ranging from *T. rubrum, T. mentagrophytes* to *M. gypseum* were examined and selected for their capability of biofilm-forming *in vitro* in our study. Due to the generally low sporulation of *T. rubrum*, only 6 out of 70 initial clinical isolates were identified capable of forming biofilm after 72 h of cultivation. Ultimately, six strains of *T. rubrum*, ten strains of *T. mentagrophytes* and three strains of *M. gypseum* capable of biofilm formation were subsequently subjected to aPDT based assays, enabling us to gain an in-depth insight into the application of aPDT against dermatophytic biofilms implicated in clinical onychopathic infections.

Photodynamic inactivation of aPDT requires the application of photosensitizer (PS) and subsequent irradiation with visible light corresponding to the specific absorption wavelength of photosensitizer (Castano et al., 2005; Plaetzer et al., 2009). A variety of PSs have been previously used in antifungal photodynamic inactivation, including MB, toluidine blue, 5-aminolevulinic acid, and so on (Cormick et al., 2009; Calzavara-Pinton et al., 2012; Dai et al., 2012). The photosensitizer MB used in our investigation has an absorption wavelength over 600 nm, which has been shown capable of exerting substantial reduction on the biofilms of dermatophytes tested. However, *T. rubrum* appeared more resistant than *T. mentagrophytes* and *M. gypseum* and the MB concentration dependence was more evident in *T. rubrum* than that of *T. mentagrophytes* and *M. gypseum*, presumably due to *T. rubrum* being equipped with abundant red pigments that may interfere the absorption of MB’s chromophore.

The effect of aPDT on the susceptibility of dermatophytic biofilms to clinically applied antifungal agents was examined and the biofilms subjected to aPDT exhibited significant reductions in SMIC80, meaning aPDT effectively increased the susceptibilities of *T. rubrum, T. mentagrophytes* and *M. gypseum* to these conventional antimicrobial drugs, including terbinafine, itraconazole, cyclopirox, and fluconazole. The mechanism underlying aPDT-induced disruption rendering biofilms more susceptible could be multi-factorial, involving photodynamic action targeting multiple cellular components, such as fractionating plasma membrane, triggering ion imbalance leading to intolerable changes in osmotic pressure and pH, and DNA breaking. The observed sensitization of dermatophytes to the antifungal agents resulted from the decreased biofilm viability in this study, however, in the future, more investigation with sub-lethal dose of aPDT could be conducted to have a better understanding of how different levels of oxidative and nitrosative stresses affecting the susceptibility of dermatophytic biofilms to antimicrobials and to facilitate the optimization of combination therapies.

In conclusion, our results suggest that photodynamic approaches hold great promise for combating the biofilm of dermatophytes involved in onychomycosis. *In vitro* photodynamic treatment with methylene blue and LED was found to be highly efficient in inactivating dermatophytic biofilms of *T. rubrum, T. mentagrophytes*, and *M. gypseum.* When aPDT applied alongside antifungal agents, it has the potential to reduce drug dosages, drug toxicity, and treatment times. Further investigation is needed to address if such efficacy could be ultimately obtained *in vivo* and it is important to optimize treatment protocols to cope with constrained drug permeation and light attenuation through nail plates in human studies.

## Author Contributions

BC, YS, and JGZ conceived and designed the study. BC, JYZ, RC, and XZ performed all the experiments. BC and XW analyzed the data and wrote the manuscript. JGZ, LZ, and XW provided the general guidance and revised the manuscript.

## Conflict of Interest Statement

The authors declare that the research was conducted in the absence of any commercial or financial relationships that could be construed as a potential conflict of interest.
